# Physiological investigation of heatable fabrics

**DOI:** 10.1186/2046-7648-4-S1-A137

**Published:** 2015-09-14

**Authors:** Edith Classen, Ferry Siegl

**Affiliations:** 1 Hohenstein Institut für Textilinnovation gGmbH, Boennigheim, Germany. TITV Greiz, Greiz, Germany

## Introduction

On the market there are various heatable fabrics with a mobile energy source and a heating power of approximately 10 watts. Examples for heatable fabrics are multilayer outdoor fabrics (pants and jackets), one layer fabrics (jackets, vests), underwear (shirts and paints), gloves, socks and shoes and accessories like a kidney belt. Up to now heatable textile fabrics are in the focus of research activities but have not been a large market. The reasons for this are several: technical problems during the life time, unfavourable system design, usage restriction caused by low life of storage battery or problems by cleaning and washing. The German research project 17708BG has the aim to investigate the clothing physiological aspects of heatable fabrics and the comfort of such fabrics.

## Methods

The thermophysiological comfort of various heatable fabrics (different underwear, outdoor jackets and trousers) can be determined by investigation of different thermophysiological parameters with the Hohenstein skin model (a sweating guarded-hotplate) and with the thermal manikin "Charlie" of whole clothing ensembles. Additional wearer trials in a climatic chamber with controlled environmental conditions are made to correlate the objective data of the Hohenstein Skin model and the manikin "Charlie" with the real wearer situation.

## Results

The investigation with the thermal manikin Charlie of different clothing systems shows the influence of heating effect depending on the position of the heating elements in the clothing. The different switching possibilities of the heating system show also different effects on the heating effect. The common thermophysiological properties e.g. the breathability and the thermal insulation can be influenced by the heating system and the integration of the heating system in the clothes. Is the heating unit performed as heat pad the material of the head pad influences the thermophysiological properties and often the breathability is low and the additional textiles layers lead to a higher thermal insulation. In underwear where the heatable zones are knitted, the influences to the thermophysiological properties are lower than in heat pads.

## Discussion

The effect of the heatable fabrics is depending on the position of the body (e.g. back, front, arms and/or arms) and the investigated fabric. The differences between different fabrics are high because various technologies are used (heat pads (in most cases removable), knitted heated or woven heated zones). The comparison of the results of the manikin tests and the wearer trials are under analysis. First results show that the effects of the heatable fabrics measured by the thermal manikin are comparable with the objective and subjective data of the wearer trials and the judgement of the heatable function can be made by the manikin tests.

## Conclusion

The results of the research project show that the physiological comfort of heatable textiles depends on the used heating technique and the dimension of the heating system. The investigation with clothing physiological measurement devices and with wearer trials show that the heatable textiles show often a low comfort and so the potential of the optimization of heatable products is high and some modification can be easily integrated in the production process. The results of the German research project (IG 17708BG) give a better understanding and more information about heatable fabrics. The results can help the producer to optimize products and to produce products with a long lifetime and high performance.
